# The association of maternal fat-soluble antioxidants in early pregnancy with gestational diabetes mellitus: a prospective cohort study

**DOI:** 10.1038/s41387-022-00227-x

**Published:** 2022-12-09

**Authors:** Yanyu Lyu, Guiyun Wang, Zhenfeng Sun, Xiaodai Cui, Qingyong Xiu, Lijun Wu

**Affiliations:** 1 Experiment Center, Capital Institute of Pediatrics, Beijing, China; 2 Department of Laboratory Medicine, Beijing Daxing Maternal and Child Care Hospital, Beijing, China; 3 Department of Obstetrics, Beijing Daxing Maternal and Child Care Hospital, Beijing, China; 4 Department of Pediatrics, Beijing Daxing Maternal and Child Care Hospital, Beijing, China; 5 Department of Epidemiology, Capital Institute of Pediatrics, Beijing, China

**Keywords:** Gestational diabetes, Gestational diabetes

## Abstract

**Introduction:**

Oxidative stress is linked to the development of gestational diabetes mellitus (GDM). Maternal antioxidant vitamins in early pregnancy may play a role in GDM occurrence. We aimed to investigate the associations of vitamins A and E in early pregnancy with the risk of GDM and to explore whether these antioxidant vitamins can be biomarkers for the early prediction of GDM.

**Methods:**

We carried out a prospective cohort study conducted in Beijing and enrolled pregnant women (*n* = 667) with vitamins A and E measurements at 9 weeks (IQR 8–10) of gestation and having one-step GDM screened with a 75-g oral glucose tolerance test between 24 and 28 weeks of gestation.

**Results:**

The vitamin A levels in early pregnancy were significantly higher in women with GDM than in those without GDM (*p* < 0.0001) and positively correlated with fasting blood glucose. In multivariate models, vitamin A levels were significantly associated with GDM (OR, 1.46; 95% CI: 1.14–1.88; *p* = 0.0032) per SD. A significant trend of risk effect on GDM risk across quartiles of vitamin A was observed (*p*_trend_ = 0.016). No significant association of serum vitamin E with GDM was observed overall. However, a noted trend of protective effect on GDM risk across quartiles of vitamin E/cholesterol ratio was observed (*p*_trend_ = 0.043). In ROC analysis, the multivariate model consisting of vitamin A and other risk factors showed the best predictive performance (AUC: 0.760; 95% CI: 0.705–0.815; *p* < 0.001).

**Conclusions:**

Higher levels of vitamin A in early pregnancy were significantly associated with an increased risk of GDM. Vitamin A has the potential to be a biomarker indicating pathogenesis of GDM.

## Introduction

Gestational diabetes mellitus (GDM) is a common complication of pregnancy defined as any degree of glucose intolerance with onset or recognition during pregnancy [1]. GDM affects ~5–17% of pregnancies, with an increasing incidence worldwide [2], and is associated with adverse long-term outcomes, including obesity, impaired glucose metabolism, and cardiovascular disease, in both the mother and offspring [3]. A recent systematic review reported that the incidence of GDM was 14.8% in China, and age, body weight, and family history of diabetes mellitus could significantly increase GDM risk [4].

Despite considerable studies have been conducted to understand the pathophysiology of GDM, the underlying mechanism is still poorly defined, especially in early pregnancy. GDM is usually diagnosed during 24–28 weeks of gestation according to a 75-g oral glucose tolerance test (OGTT) [5]. Thus, identifying modifiable factors in early pregnancy is desired for GDM prediction and early intervention.

Many studies have indicated that oxidative stress can cause hyperglycemia, intrauterine growth restriction, and miscarriage [6]. An imbalance of oxidative stress in GDM patients leads to damage to vascular and pancreatic β-cells and affects pregnancy outcomes [7]. In women with GDM, the radical scavenger function is impaired, and there is an overproduction of free radicals [8]; meanwhile, the levels of several oxidative stress markers are higher [9]. Since oxidative stress is a known cause of cellular damage by interfering with the state of proteins, lipids, and DNA, and has been implicated in the pathogenesis of metabolic and hypertensive disorders of pregnancy, including GDM [10, 11], we hypothesized that insufficient antioxidant defenses in early pregnancy may therefore predispose women to GDM.

Fat-soluble vitamins A and E are essential micronutrients in the human body and have antioxidant properties that can block the initiation of free radical formation and inactivate free radicals [12–14]. Both vitamins play critical roles in maternal health and fetal development [15]. Vitamin A plays an important role in immunity, cell proliferation, and differentiation, embryonic development, and metabolic disease prevention and causation [16, 17]. Vitamin A deficiency or excessive vitamin A could both affect embryonic development. Vitamin E is an essential vitamin for maintaining metabolic function and scavenging free radicals. Vitamin E deficiency leads to placental aging, premature birth, and placental abruption.

Several studies have reported lower levels of fat-soluble antioxidant vitamins in women with GDM [18, 19]. However, most studies measured antioxidant levels in mid- or late pregnancy and had inconsistent findings [20, 21]. Determining the levels of antioxidants earlier in pregnancy would elucidate whether antioxidant deficit predicts GDM diagnosis and could be considered in future intervention studies. Given that little work was done investigating the association of antioxidant vitamin status in early pregnancy and subsequent GDM and had heterogeneous findings, we aimed to estimate whether fat-soluble antioxidant vitamins in early pregnancy are associated with GDM occurrence and can be potential biomarkers to predict GDM.

## Materials and methods

### Study design and study population

We carried out this study with the Maternal and Infant Health birth cohort that aimed to investigate the association between maternal obesity and psychological status on infant growth and neurocognitive development. We recruited singleton pregnancies in the first trimester (<13 gestational weeks) in Beijing Daxing Maternal and Child Care Hospital from November 2016 to December 2017. Pregnant women who were at least 18 years of age, planned to deliver infants, and received child health care for their infants at the site hospital were eligible to participate. A total of 982 singleton pregnancies were enrolled at the first prenatal visit during early pregnancy, and 805 were followed to deliver single live births. Three women who had preexisting diabetes were excluded. Of the 802 pregnant women, 667 subjects were obtained for both having measurements of vitamin A and vitamin E in early pregnancy and having one-step GDM screened with OGTT between 24 and 28 weeks of gestation. A total of 667 pregnancies were included in the final analysis. This prospective cohort study was approved by the Ethical Committee of Capital Institute of Pediatrics (SHERLL-2016034), and written informed consent was obtained from each subject before recruitment.

### Vitamin A and vitamin E measurement

Fasting blood samples were obtained from the pregnant women at the first prenatal visit in early pregnancy as part of routine prenatal screening examination. All collections were finished between 8:00 and 10:00 in the morning. The serum samples were stored in cold chain and transported to Beijing Harmony Health Medical Diagnostic Laboratory for quantitative detection on the same day. Serum vitamin A (retinol) and vitamin E (α-tocopherol) were measured by high-performance liquid chromatography (Agilent, USA). The standard substance was purchased from the American Sigma Company. The Westgard multirule quality control procedure was adopted to help analyze whether an analytical run was in control. Routine blood biochemical indicators, including fasting blood glucose (FBG) and total cholesterol, were tested according to standard detection methods using an automatic biochemical analyzer (Olympus Chemistry Analyzer AU640; Olympus Optical Co., Ltd, Tokyo, Japan). Because the ratio of vitamin E to cholesterol has been reported to be the most useful measurement of vitamin E status in blood [22], we also examined the exposure of the vitamin E/cholesterol ratio and risk of GDM.

### Diagnosis of GDM

Between 24- and 28 weeks during pregnancy, pregnant women visited the hospital to diagnose GDM using OGTT. Venous blood samples were collected at 0, 1, and 2 h after a 75-g glucose load. If one or more of the blood glucose levels were met or exceeded in the 75-g OGTT: 0 h (fasting) ≥5.10 mmol/L; 1 h ≥ 10.00 mmol/L; and 2 h ≥ 8.50 mmol/L, women were diagnosed with GDM according to the recommendations of the International Association of the Diabetes and Pregnancy Study Groups Consensus Panel [23].

### Data collection

Baseline characteristics and sociodemographic information were obtained by a standardized self-report questionnaire. The pre-pregnancy body mass index (BMI) was calculated through self-reported pre-pregnancy weight and height. The levels of FBG and total cholesterol at enrollment, the blood glucose levels of OGTT, and gestational weight gain (GWG) in early pregnancy (before 18 gestational weeks) were collected from the hospital electronic information system (HIS). The information on vitamins supplementation was collected in the questionnaire survey at enrollment although we paid more attention to the internal exposure of vitamin concentrations represented by serum concentration.

### Statistical analysis

Continuous data were summarized as the means (standard deviation [SD]) or medians (interquartile ranges [IQR]) due to their distribution, and categorical data were presented as percentages. Characteristics of the study population were compared between GDM and non-GDM groups using the *χ*^2^ test for categorical variables and Student’s *t* test or nonparametric Wilcoxon test for continuous variables as appropriate. As vitamin A and vitamin E had a skewed distribution, we transformed each value to a *z* score (units correspond to SD in the non-GDM population). Vitamin A and vitamin E levels were analyzed both as continuous variables (*z* score) and divided into quartiles to determine possible nonlinear relationships. In the multivariate analysis, we did not impute the missing data on family history of diabetes for 4 (0.6%) pregnant women, GWG in early pregnancy for 22 (3.3%), and FBG in early pregnancy for 3 (0.4%). These pregnant women were ruled out automatically when running the regression that included the covariate of family history of diabetes, GWG in early pregnancy, or FBG in early pregnancy. We assessed the association between each antioxidant vitamin and GDM through multivariable logistic regression models with adjustments for maternal age, family history of diabetes, GWG in early pregnancy, FBG at enrollment, and pre-pregnancy BMI. We used the *z* score of the vitamin E/cholesterol ratio and quartiles of the vitamin E/cholesterol ratio as exposures to investigate the association with GDM risk. In sensitivity analyses, we assessed the associations between each of the antioxidant biomarkers and GDM risk after excluding participants who had taken multivitamins in early pregnancy. Correlation analysis was used to evaluate the relationship of vitamin A or vitamin E with FBG at enrollment and in OGTT and 1-h and 2-h serum glucose in OGTT. Receiver operator characteristic (ROC) analysis was performed, and the area under the curve (AUC) was used to evaluate their predictive capabilities. All statistical analyses were performed using R statistical software version 3.5.1 (R Project for Statistical Computing; http://www.r-project.org). *p* < 0.05 was used to indicate statistically significant differences.

## Results

**Fig. 1:**
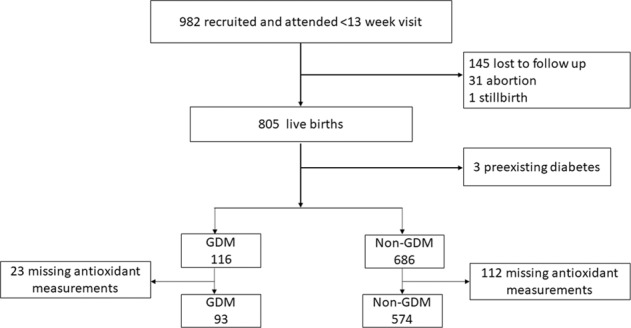
Flow diagram of study population. GDM gestational diabetes mellitus.

The flow diagram of the study population is shown in Fig. 1. A total of 667 pregnant women were included in the analysis. Ninety-three of them developed GDM (13.94%). The median serum concentration of vitamin A was 0.47 (IQR 0.41–0.53) mg/L and that of vitamin E was 10.6 (IQR 9.3–12.1) mg/L.

The characteristics of women with GDM and non-GDM are described in Table 1. Women who developed GDM had higher means of pre-pregnancy BMI and GWG in early pregnancy and were more likely to have a family history of diabetes than non-GDM women. Mean FBG levels in early pregnancy were higher in women with GDM. Vitamin A was significantly higher in pregnancies with GDM than in those without GDM. However, no significant difference in vitamin E levels was observed between women with GDM and non-GDM.

**Table 1:** Basic characteristics of women with GDM and non-GDM (*N* = 667).

Maternal characteristic	GDM (*n* = 93)	Non-GDM (*n* = 574)	*p* value
Age (years)	30.83 ± 4.07	29.97 ± 3.74	**0.042**
Gestational age at enrollment (weeks)	9.1 (8.0–10.7)	9.4 (8.3–10.4)	0.93
Pre-pregnancy BMI (kg/m^2^)	24.16 ± 3.92	22.34 ± 3.51	**<0.0001**
Nulliparity	48 (51.6)	305 (53.1)	0.78
Family history of diebetes, %	19 (20.4)	71 (12.5)	**0.037**
GWG in early pregnancy (kg/wk)	0.227 (0.100–0.375)	0.172 (0.060–0.272)	**0.010**
Preexisting chronic conditions			
Thyroid disease, %	1 (1.1)	18 (3.1)	0.27
Anemia, %	4 (4.3)	43 (7.5)	0.26
Allergy disease, %	2 (2.2)	17 (3.0)	0.66
Blood samples at enrollment			
FBG (mmol/L)	5.07 ± 0.43	4.82 ± 0.34	**<0.0001**
Retinol (mg/L)	0.51 (0.45–0.58)	0.46 (0.41–0.52)	**<0.0001**
α-Tocopherol (mg/L)	10.9 (9.5–12.2)	10.6 (9.3–12.1)	0.50

Those highlighted in bold indicate that differences were statistically significant Missing information: family history of diabetes for 4, GWG in early pregnancy for 22, and FBG in early pregnancy for 3.

*GDM* gestational diabetes mellitus, *BMI* body mass index, *GWG* gestational weight gain, *FBG* fasting blood glucose.

**Fig. 2:**
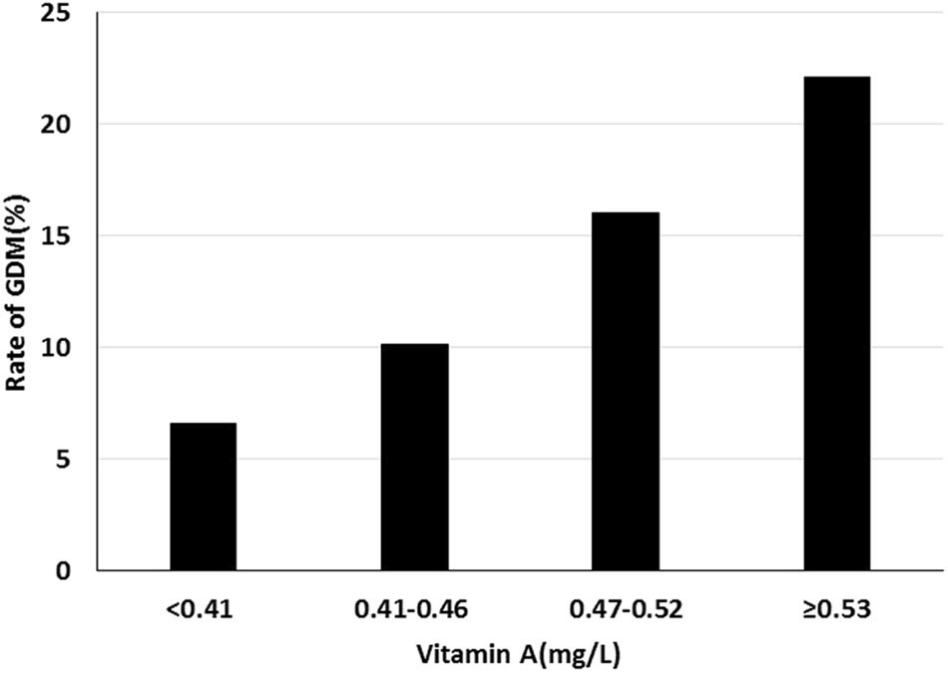
Association of maternal vitamin A levels in early pregnancy with GDM. GDM gestational diabetes mellitus.

Univariate and multivariate associations of maternal vitamins A and E levels with the odds of GDM are shown in Table 2. According to the crude logistic regression analysis using the standardized *z* score as the main predictor variable, vitamin A levels were positively associated with the risk of GDM. After adjustment for maternal age, family history of diabetes, GWG in early pregnancy, FBG in early pregnancy, and pre-pregnancy BMI, the association remained significant (odds ratio [OR], 1.46, 95% confidence interval [CI]: 1.14–1.88, *p* = 0.0032). In the analysis of vitamin A in quartiles, women with the highest vitamin A levels (quartile 4) were associated with two-fold higher odds of GDM (OR, 2.25, 95% CI: 1.02–4.98, *p* = 0.046) than women in the lowest quartile (quartile 1). An obvious increased risk trend across different levels of vitamin A groups with GDM risk was observed (*p*_trend_ = 0.016), and Fig. 2 also exhibits this trend. There was no significant association of the standardized *z* score or quartiles of vitamin E in early pregnancy with the risk of GDM. For the vitamin E/cholesterol ratio balance and GDM, the standardized *z* score of the vitamin E/cholesterol ratio was not significantly associated with GDM, while it showed a marginally significant trend (*p*_trend_ = 0.043).

**Table 2:** Univariate and multivariate associations of maternal antioxidant vitamins A and E with odds of GDM.

	GDM, n/N (%)	Model 1			Model 2		
		OR	95% CI	*p* value	OR	95% CI	*p* value
Retinol continuous, *z* score		1.75	1.42–2.17	**<0.0001**	1.46	1.14–1.88	**0.0032**
Retinol quartiles (mg/L)							
Q1	10/152 (6.58)		Reference			Reference	
Q2	18/178 (10.11)	1.60	0.71–3.57	0.25	1.19	0.51–2.73	0.30
Q3	25/156 (16.03)	2.71	1.25–5.86	0.15	1.89	0.84–4.23	0.27
Q4	40/181 (22.10)	4.03	1.94–8.37	**0.0001**	2.25	1.02–4.98	**0.046**
Trend test				**<0.0001**			**0.016**
α-Tocopherol continuous, z-score		1.20	0.99–1.46	0.066	1.21	0.97–1.51	0.094
α-Tocopherol quartiles (mg/L)							
Q1	21/157 (13.38)		Reference			Reference	
Q2	21/162 (12.96)	0.97	0.50–1.85	0.69	0.80	0.40–1.62	0.54
Q3	26/178 (14.61)	1.11	0.60–2.06	0.76	0.93	0.48–1.82	0.95
Q4	25/170 (14.71)	1.12	0.60–2.09	0.73	0.95	0.47–1.92	0.87
Trend test				0.64			0.99
α-Tocopherol/CHO continuous, *z* score		1.00	0.80–1.24	0.99	1.04	0.82–1.31	0.74
α-Tocopherol/CHO quartiles							
Q1	31/166 (18.67)		Reference			Reference	
Q2	23/166 (13.86)	0.70	0.39–1.26	0.97	0.79	0.42–1.50	0.57
Q3	21/166 (12.65)	0.63	0.35–1.15	0.63	0.59	0.31–1.15	0.43
Q4	18/166 (10.84)	0.53	0.28–1.00	0.19	0.52	0.26–1.05	0.20
Trend test				**0.041**			**0.043**

Model 1 was a univariate model. Model 2 was adjusted for maternal age, a family history of diabetes, pre-pregnancy BMI, GWG in early pregnancy, FBG at enrollment. Those highlighted in bold indicate that the associations showed statistical significance. Missing information: cholesterol 3.

*GDM* gestational diabetes mellitus, *OR* odds ratio, *CI* confidence interval, *CHO* cholesterol.

As shown in Table 3, the vitamin A concentration in early pregnancy was positively correlated with FBG in early pregnancy (*r* = 0.090, *p* < 0.05) and highly correlated with OGTT FBG, OGTT 1-h, and 2-h serum glucose levels, with correlation coefficient *r* values of 0.22, 0.18 and 0.24, respectively (all *p* < 0.0001). The vitamin E concentrations in early pregnancy were negatively correlated with FBG in early pregnancy (*r* = −0.085, *p* < 0.05). No significant correlations were found between the vitamin E concentration and OGTT FBG.

**Table 3:** Pearson correlations of retinol and α-tocopherol with FBG at enrollment, OGTT FBG, OGTT 1-h and 2-h blood glucose levels in women with non-GDM (*n* = 574).

	Retinol	α-Tocopherol	FBG at enrollment	OGTT 1-h blood glucose	OGTT 2-h blood glucose
α-Tocopherol	0.33***				
FBG at enrollment	0.090*	−0.085*			
OGTT FBG	0.22***	0.018	0.37***		
OGTT 1-h blood glucose	0.18***	0.11**	0.20***	0.43***	
OGTT 2-h blood glucose	0.24***	0.12**	0.17***	0.30***	0.57***

*FBG* fasting blood glucose, *OGTT* oral glucose tolerance test, *GDM* gestational diabetes mellitus.

*p < 0.05.

**p < 0.01.

***p < 0.0001.

In ROC analysis, with an AUC of 0.653, the vitamin A levels showed a significantly discriminatory ability to predict GDM compared with maternal age (AUC, 0.554; *p* = 0.019) and a family history of diabetes (AUC, 0.540; *p* = 0.0022), but were similar to pre-gestational BMI (AUC, 0.643; *p* = 0.80), GWG in early pregnancy (AUC, 0.584; *p* = 0.13) and FBG levels in early pregnancy (AUC, 0.679; *p* = 0.56) (Supplementary Table 1).

**Fig. 3:**
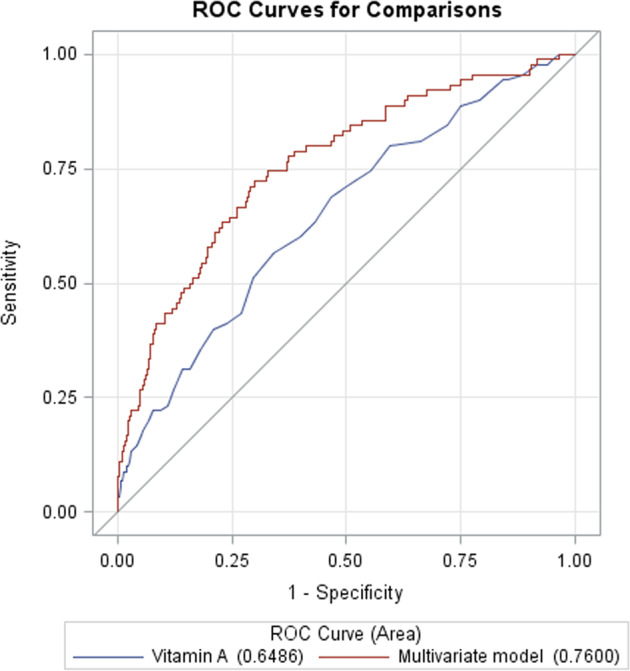
ROC curves were utilized to evaluate the accuracy of the vitamin A levels in early pregnancy to predict GDM. ROC receiver operator characteristic.

Furthermore, the multivariate model consisting of the vitamin A levels, maternal age, a family history of diabetes, GWG in early pregnancy, FBG levels in early pregnancy, and pre-pregnancy BMI showed the best predictive performance (AUC, 0.760; 95% CI: 0.705–0.815; *p* < 0.001). Compared to the model including vitamin A only (AUC, 0.649; 95% CI: 0.587–0.710; *p* < 0.001), there was a significant statistical difference in the AUC between the multivariate model and vitamin A alone (difference 0.111; 95% CI: 0.053–0.170; *p* = 0.0002) (Fig. 3).

## Discussion

In this prospective cohort, we found that higher levels of vitamin A in early pregnancy were significantly associated with an increased risk of GDM. The vitamin E levels were not significantly associated with GDM overall, whereas a trend of reduced risk of GDM across quartiles of vitamin E/cholesterol ratio was observed. Positive correlations of vitamin A with FBG in early pregnancy, FBG, 1-h, and 2-h serum glucose levels in OGTT supported our findings.

To our knowledge, our study provides the first evidence that a higher serum vitamin A concentration in early pregnancy might confer an increased GDM risk. Previous studies indicated that oxidative stress might be involved in the pathogenesis of GDM. Fat-soluble vitamins A and E are essential micronutrients for the human body with antioxidant properties that can prevent the initiation of free radical formation and inactivate free radicals and play critical roles in maternal health. Because GDM is usually diagnosed during 24–28 weeks of gestation, identifying modifiable factors in early pregnancy is important for GDM prediction and early intervention. Thus, we assessed the associations of maternal vitamins A and E levels in early pregnancy with GDM occurrence and explored whether those can be potential biomarkers to predict GDM. Although our results for vitamin A were contrary to the study hypothesis, increased levels of vitamin A have a relevant role in the development of GDM, suggesting new insights into the pathogenesis of GDM.

Our results were comparable to those of a previous retrospective study including 2116 Chinese pregnant women, which reported that there were higher vitamin A levels and lower vitamin E levels in early pregnancy in the GDM group than in the non-GDM group, but they were not identified as independent factors for GDM in multiple logistic regression analysis [24]. That results are not very strong because the study did not specify which confounders were controlled. Cohen et al. revealed that the retinol concentrations in mid-pregnancy were positively associated with the risk of subsequent preeclampsia, although most antioxidants were inversely associated with preeclampsia [25]. In contrast, Fruscalzo et al. reported that low retinol plasma concentrations in the first trimester were predictive of the development of insulin-treated GDM after adjusting for confounding factors [26]. A recent study from China showed the opposite results as ours, but it did not further study the associations of lower vitamin A levels and higher vitamin E levels with GDM risk [20]. Parast et al. [27] reported that antioxidant capacity was lower in women with GDM, possibly related to lower intakes of vitamin E and zinc. However, the latter two studies had difficulty establishing causal effects between antioxidants and GDM because both measurements of antioxidants and diagnosis of GDM were achieved at 24–28 gestational age.

Previous studies have indicated that the vitamin nutrient pattern diet, characterized as the consumption of a diet rich in vitamin A, carotene, vitamin B2, vitamin B6, vitamin C, dietary fiber, folate, calcium, and potassium, is associated with decreased GDM risk [28]. Wang et al. also demonstrated that higher vitamin A intake from animal-derived food is associated with a decreased risk of GDM [29]. Although we lack investigation on dietary intake, antioxidant vitamin levels in circulation might be more informative than dietary intake because they have more accurate measurements, taking into account the absorption of vitamins, which reflect antioxidant vitamin levels closer to biological function.

The mechanisms by which vitamin A may confer the risk of GDM are not well understood. Retinol and its active derivatives can undertake many physiological functions. Vitamin A deficiency has long been known to be deleterious to the mother and fetus, whereas an excess of vitamin A can exert toxic and teratogenic effects in early pregnancy [17].

Retinol-binding protein 4 (RBP4) is the only specific transport protein for retinol, traveling as an RBP4-retinol complex in the circulation bound to the carrier protein transthyretin (TTR). Plasma RBP4 levels in the first trimester, independent of metabolic risk factors, are associated with an increased risk of GDM in pregnant women [30]. Investigation of the RBP4 levels in the first trimester and analysis of the relationships between the RBP4 levels and the risk of GDM will be carried out in our future study.

In addition, liver dysfunction occurs in up to 3% of pregnancies and results in significant changes in some laboratory values, including alkaline phosphatase, triglycerides, and cholesterol [31]. Even in normal pregnancy, many physiological and hormonal changes may occur, some of which are similar to those in women with liver disease [32]. It is well-established that hepatocytes play important roles in the storage and metabolism of vitamin A [33]. Chen et al. observed that the levels of an indicator of mild liver dysfunction, g-glutamyl transferase, were higher in the GDM group and assumed that women with GDM might have concurrent mild liver dysfunction [34]. Since vitamin A is stored and metabolized in hepatocytes, it is speculated that pregnancy may have a possible effect on vitamin A levels. However, this is not sufficient to explain the higher levels of vitamin A in the GDM group in our study.

Prenatal multivitamins intake is an important dietary exposure that is related to vitamin A and vitamin E levels. There were 73 women in the sample who had been taking multivitamins, containing vitamin A and vitamin E, for 6.0 (2.7–11.7) weeks (median, IQR) before blood sample collection in early pregnancy. We compared vitamin A and vitamin E levels, and GDM occurrence in the multivitamins group and the no multivitamins group (Supplementary Table 2). Both vitamin A and vitamin E levels were significantly higher in the multivitamins group than in the no multivitamins group. The rates of GDM were not significantly different between these two groups. Furthermore, a sensitivity analysis excluding those with multivitamins intake in early pregnancy was conducted. We found that vitamin A levels were still positively associated with the risk of GDM. However, in the analysis of vitamin A in quartiles, compared with pregnant women who had the lowest 25% values of vitamin A, those with the highest 25% values of vitamin A were not significantly associated with GDM (Supplementary Table 3). The coefficients did not change much, and the direction of association did not change. The increased risk trend across different levels of vitamin A groups with GDM risk was still obvious. This might be related to the reduced sample size and the original effect between them was not strong. In general, the sensitivity analysis resulted in similar odds ratios and did not alter the major conclusions.

Currently, there is no clinical application of circulating biomarkers for the accurate prediction of GDM. As a potential biomarker for GDM, the predictive power of vitamin A (AUC 0.653, *p* < 0.001) was similar to FBG in early pregnancy in our study. A combination of vitamin A and FBG in early pregnancy, demographic information (maternal age and family history of diabetes), and common clinical characteristics (pre-gestational BMI and GWG in early pregnancy) could distinguish later GDM from healthy pregnant women (AUC 0.760, *p* < 0.001). Hou et al. constructed a model consisting of pre-pregnancy BMI, RBP4, n-acetylaspartic acid, and C16:1 (cis-7) and achieved the best discrimination (AUC 0.751, 95% CI: 0.693–0.809, *p* < 0.001) using peripheral blood collected from Chinese women at ~12 weeks of gestation [35]. Huang et al. found that the mean platelet volume (AUC 0.577, 95% CI: 0.533–0.621) and plateletcrit (AUC 0.628, 95% CI: 0.582–0.674) in early pregnancy were potential indicators in predicting GDM [36]. In the future, the development of multiple clinical biomarkers and other variables that can be measured conveniently could improve the discriminative abilities of GDM.

In our study, there was no significant association between vitamin E levels and GDM, whereas a noted trend of protective effect on GDM risk across quartiles of vitamin E/cholesterol ration was observed. Most studies directly investigated the relationship of vitamin E with GDM. A recent systematic review reported that vitamin E levels were significantly lower in women with GDM than in healthy pregnant women [37]. But given the nature of the observational studies in this systematic review, a causal relationship between vitamin E and GDM could not be drawn. The ratio of vitamin E (α-tocopherol)/cholesterol is a clear marker of the relative insufficiency or excess of this antioxidant regarding the efficient functioning of its action. Cohen et al. reported that a higher ratio of α-tocopherol to cholesterol, but not α-tocopherol, was significantly associated with a reduced risk of early-onset preeclampsia [25]. Since preeclampsia and GDM are both pregnancy complications and are related to oxidative stress [10], the protective effect of vitamin E for these diseases is reasonable. However, the opposite effect of vitamin A raises an important question regarding the potential mechanism or causality for the development of GDM and hypertensive disorders of pregnancy. The role of oxidative stress, especially in obstetrics-related conditions, cannot well explain its pathogenesis and pathophysiology.

This study has several limitations. First, we did not measure RBP4 and TTR levels, which are bound to retinol in circulation. Second, our study did not have information on dietary details and physical activities. These confounding factors were not excluded. Third, the study recruitment was in a special hospital for women and children in a district of Beijing. Although two-thirds of the study population was not born in Beijing and came from all over the country, caution is needed for the generalizability of our results to a wider population. Fourth, our study was not the primary study in this prospective cohort and did not have a prespecified power calculation. Our sample size might be not enough for some aspects of the study.

In conclusion, our results indicated that higher vitamin A levels in early pregnancy were significantly associated with the risk of GDM. There was no significant association of vitamin E levels in early pregnancy with GDM, but a trend of decreased GDM risk across quartiles of vitamin E/cholesterol was observed. The vitamin A concentrations might be helpful for the early identification of pregnancy at risk of developing GDM.

## Supplementary information

Supplementary Tables

## Data Availability

The datasets generated during and/or analyzed during the current study are available from the corresponding author upon reasonable request.
