# Supplementary material for: The association of maternal fat-soluble antioxidants in early pregnancy with gestational diabetes mellitus: a prospective cohort study
Source: Nutr Diabetes. 2022 Dec 9;12:49. doi: 10.1038/s41387-022-00227-x (PMC9734187; doi:10.1038/s41387-022-00227-x)
Supplement: Supplementary file 1 — Supplementary Tables [file 41387_2022_227_MOESM1_ESM.docx]

**Supplementary Table 1.** Risk factors in early pregnancy prediction of GDM in ROC analysis.

| Risk factors | AUC | 95%CI | p-value |
| --- | --- | --- | --- |
| Maternal age | 0.554 | 0.489-0.619 | **0.019** |
| Family history of diabetes | 0.540 | 0.497-0.583 | **0.0022** |
| Pre-gestational BMI | 0.643 | 0.582-0.704 | 0.80 |
| GWG in early pregnancy | 0.584 | 0.518-0.650 | 0.13 |
| FBG at enrollment | 0.679 | 0.619-0.740 | 0.56 |
| Retinol at enrollment | 0.653 | 0.593-0.713 | Reference |

Those highlighted in bold indicate that the differences were statistically significant.

GDM, Gestational diabetes mellitus; ROC, Receiver operator characteristic; AUC, Area under the curve; CI, Confidence interval; BMI, Body mass index; GWG, Gestational weight gain; FBG, Fasting blood glucose.

**Supplementary Table 2.** Distribution of maternal vitamin A and vitamin E levels in early pregnancy, and GDM rates in the multivitamins group and the no multivitamins group.

|  |  | Multivitamins (n=73) |  | No multivitamins (n=594) | | p-value | |  |
| --- | --- | --- | --- | --- | --- | --- | --- | --- |
|  |  | Median (IQR) or N (%) |  | Median (IQR) or N (%) |  | |  | |
| Vitamin A (mg/L) | | 0.48 (0.43-0.56) |  | 0.46 (0.41-0.53) |  | | **0.033** | |
| Vitamin E (mg/L) | | 11.00 (10.20-12.00) |  | 10.60 (9.20-12.10) |  | | **0.011** | |
| GDM | | 11 (15.07) |  | 82 (13.80) | | 0.769 | |  |

GDM, gestational diabetes mellitus; IQR, interquartile range.

Those highlighted in bold indicate that the differences were statistically significant.

**Supplementary Table 3.** Univariate and multivariate associations of maternal antioxidant vitamins A and E with odds of GDM in the no multivitamins group.

|  | GDM, n/N (%) | Model 1 | | |  | Model 2 | | |
| --- | --- | --- | --- | --- | --- | --- | --- | --- |
|  |  | OR | 95% CI | p-value |  | OR | 95% CI | p-value |
| Retinol continuous, z-score |  | 1.84 | 1.46-2.31 | **<0.0001** |  | 1.50 | 1.14-1.98 | **0.0037** |
| Retinol quartiles (mg/L) |  |  |  |  |  |  |  |  |
| Q1 | 9/141 (6.38) |  | Reference |  |  |  | Reference |  |
| Q2 | 15/157 (9.55) | 1.55 | 0.66-3.66 | 0.202 |  | 1.14 | 0.47-2.79 | 0.3023 |
| Q3 | 23/142 (16.20) | 2.83 | 1.26-6.37 | 0.1418 |  | 1.86 | 0.79-4.38 | 0.2904 |
| Q4 | 35/154 (22.73) | 4.31 | 1.99-9.35 | **0.0001** |  | 2.20 | 0.94-5.16 | 0.0682 |
| Trend test |  |  |  | **<0.0001** |  |  |  | **0.0276** |
| α-Tocopherol continuous, z-score |  | 1.19 | 0.97-1.45 | 0.095 |  | 1.19 | 0.94 1.51 | 0.1417 |
| α-Tocopherol quartiles (mg/L) |  |  |  |  |  |  |  |  |
| Q1 | 21/150 (14.00) |  | Reference |  |  |  | Reference |  |
| Q2 | 18/145 (12.41) | 0.87 | 0.44-1.71 | 0.577 |  | 0.67 | 0.32-1.40 | 0.405 |
| Q3 | 21/146 (14.38) | 1.03 | 0.54-1.98 | 0.8055 |  | 0.80 | 0.39-1.63 | 0.9149 |
| Q4 | 22/153 (14.38) | 1.03 | 0.54-1.97 | 0.804 |  | 0.83 | 0.40-1.73 | 0.9443 |
| Trend test |  |  |  | 0.8074 |  |  |  | 0.7279 |
| α-Tocopherol/CHO continuous, z-score* |  | 0.98 | 0.78-1.24 | 0.8731 |  | 1.03 | 0.81-1.33 | 0.7969 |
| α-Tocopherol/CHO quartiles* |  |  |  |  |  |  |  |  |
| Q1 | 29/151 (19.21) |  | Reference |  |  |  | Reference |  |
| Q2 | 21/152 (13.82) | 0.67 | 0.37-1.25 | 0.8852 |  | 0.77 | 0.39-1.51 | 0.4756 |
| Q3 | 18/146 (12.33) | 0.59 | 0.31-1.12 | 0.6398 |  | 0.53 | 0.26-1.07 | 0.3546 |
| Q4 | 14/142 (9.86) | 0.46 | **0.23-0.91** | 0.1316 |  | 0.46 | **0.21-0.99** | 0.1725 |
| Trend test |  |  |  | **0.0213** |  |  |  | **0.0242** |

GDM, gestational diabetes mellitus; OR, odds ratio; CI, confidence interval; CHO, cholesterol.

Model 1 was a univariate model.

Model 2 was adjusted for maternal age, a family history of diabetes, pre-pregnancy BMI, GWG in early pregnancy, FBG at enrollment.

Those highlighted in bold indicate that the associations showed statistical significance.

*Missing information: cholesterol 3.
